# Estimating the Specialist Surgical Workforce Density in South Africa

**DOI:** 10.5334/aogh.3480

**Published:** 2021-08-16

**Authors:** Ritika Tiwari, Usuf Chikte, Kathryn M. Chu

**Affiliations:** 1 Stellenbosch University, ZA

## Abstract

**Background:**

South Africa is an upper middle-income country with inequitable access to healthcare. There is a maldistribution of doctors between the private and public sectors, the latter which serves 86% of the population but has less than half of the human resources.

**Objective:**

The objective of this study was to estimate the specialist surgical workforce density in South Africa.

**Methods:**

This was a retrospective record-based review of the specialist surgical workforce in South Africa as defined by registration with the Health Professionals Council of South Africa for three cadres: 1) surgeons, and 2) anaesthesiologists, and 3) obstetrician/gynaecologists (OBGYN).

**Findings:**

The specialist surgical workforce in South Africa doubled from 2004 (N = 2956) to 2019 (N = 6144). As of December 2019, there were 3096 surgeons (50.4%), 1268 (20.6%) OBGYN, and 1780 (29.0%) anaesthesiologists. The specialist surgical workforce density in 2019 was 10.5 per 100,000 population which ranged from 1.8 in Limpopo and 22.8 per 100,000 in Western Cape province. The proportion of females and those classified other than white increased between 2004–2019.

**Conclusion:**

South Africa falls short of the minimum specialist workforce density of 20 per 100,000 to provide adequate essential and emergency surgical care. In order to address the current and future burden of disease treatable by surgical care, South Africa needs a robust surgical healthcare system with adequate human resources, to translate healthcare services into improved health outcomes.

## Introduction

Approximately five billion people lack access to emergency and essential surgical care across the globe and 143 million additional surgical procedures are needed in low and middle income countries (LMICs) each year to save lives and prevent disability [1]. However, only 6% of surgical procedures occur in the poorest countries, where one-third of world’s population resides [1]. The lack of surgical care takes a serious human and economic toll and can lead to acute, life-threatening complications. In other instances, poor-quality care results in chronic disabilities that make productive employment impossible and impose a burden on family members and society.

Surgery is a cross-cutting service that includes procedures taking place in an operating room under anaesthesia. As such, the surgical workforce includes a broad set of specialists including surgeons, anaesthesiologists, and obstetrician/gynaecologists (OBGYN). Africa has a dire shortage of healthcare workers and the surgical workforce is no exception [2]. Only 12% of the global surgical workforce are located in Africa and southeast Asia, where a third of the world’s population lives [3].

In 2015, the Lancet Commission on Global Surgery (LCGS) released the landmark initial report Global Surgery 2030 which described the role of surgical and anaesthesia care in improving the health of individuals. The report developed core indicators to improve access to timely and safe surgical care in each country including a minimum specialist surgical workforce density of 20 per 100,000 persons in each country [1]. This target of a minimum specialist surgical workforce density addresses key SDG target 3.c on an increase in health financing and in the recruitment, development, training and retention of the health workforce, especially in low-and middle-income countries (LMICs) to improve universal health coverage (UHC) [4].

This minimum density was determined by plotting estimates of national specialist surgical workforce density against maternal survival and identifying the point where the curve in the improvement of maternal survival flattened [5]. Most African countries fall below this minimum density [6].

South Africa is an upper middle-income country (UMIC) with inequitable access to healthcare. There is a maldistribution of doctors between the private and public sectors, the latter which serves 86% of the population but has less than half of the human resources [6]. In 2014, the World Bank estimated the minimum surgical workforce density to be 11 per 100,000, however this was based on modelling and not primary data. Previous studies have attempted to measure specific cadres but never the entire surgical workforce [7,8,9,10]. The objective of this study was to estimate the surgical workforce density in South Africa. These findings can be used to characterize current gaps and inequities in the workforce as well help forecast the pipeline of surgical providers needed to improve access timeous and safe surgical care.

## Methods

### Study population and design

Database from the Health Professions Council of South Africa (HPCSA) was procured up to December 2019. Under its ambit the HPCSA has established 12 Professional Boards to provide for control over the education, training and registration for practicing of health professions registered under the Health Professions Act. HPCSA thus ensures that practitioners uphold and maintain professional and ethical standards within the health professions thus protecting the public and guiding the professions. This was a retrospective record-based review of the surgical workforce in South Africa as defined by registration with the Health Professionals Council of South Africa (HPCSA) of three cadres: 1) surgeons, and 2) anaesthesiologists, and 3) obstetrician/gynaecologists (OBGYN). Surgeons including those registered in the following subspecialty categories: general surgery, vascular surgery, paediatric surgery, trauma surgery, gastroenterology, ophthalmology, plastic and reconstructive surgery, orthopaedic surgery, cardiothoracic surgery, and neurosurgery. LCGS does not describe specific minimum densities for each surgical subspecialty category but in South Africa, general and orthopaedic surgeons would be expected to be more ubiquitous because they are found at all hospital levels while the other subspecialties work at tertiary hospitals almost exclusively. The category “critical care” was excluded (***Table 1***) because these practitioners may care for critically ill patients with conditions other than surgical ones [11].

**Table 1:** South African Specialist Surgical Workforce Categories (as per HPCSA, 2019).


SPECIALITY	SUB-SPECIALITY

Surgery	General Surgery

	Vascular Surgery

	Paediatric Surgery

	Trauma Surgery

	Gastroenterology

	Ophthalmology

	Plastic and Reconstructive Surgery

	Orthopaedic Surgery

	Cardiothoracic Surgery

	Neurosurgery

	Critical Care*

Obstetrics and Gynaecology	

Anaesthesiology	


* Excluded from analysis.

### Data collection

De-identified data were collected from the HPCSA database from 2004–2019 using a standardized data collection sheet. A similar approach was adopted as in previous studies [12,13,14,15]. Data included annual volume, province, speciality type as well as the demographic profile by sex, population group and age. The term population group was used in line with the definitions in the Population Registration Act (Act No. 30 of 1950) which previously classified South African citizens into five major categories: “white,” Chinese,” “coloured,” “Indian” and “black” [16]. The “Indian” and “Chinese” categories were combined “Asian” due to small numbers. Coloured was a term used officially in South Africa to designate persons of mixed “race.” Although the legislation was repealed in 1991, the population categories still form the basis of some official policies and statistics aimed at redressing past economic imbalances. The population categories are required in some instances, such as by the HPCSA and the Department of Higher Education and Training. These categories are also used as a measure to monitor redress in the education and training of health professionals amongst population groups who were previously denied access to such training in terms of apartheid legislation. Geographic location was categorized by province, unknown (no province listed) and foreign (out of country).

### Data analysis

Data were entered into a Microsoft Excel spreadsheet and analysed using the Statistical Package for the Social Sciences (SPSS version 22.0) [17]. Frequency distributions, cross-tabulations and graphical representations were used as descriptive statistical methods. The absolute number and demographic profile of each of the three major cadres (surgeons, anaesthesiologists, and OBGYN) included those in unknown and foreign categories. For the geographic profile and the specialist workforce density calculations, unknown and foreign categories were excluded. The workforce density was calculated in five-year intervals for 2004–2019 and population group and gender proportions were calculated for 2004 and 2019 to identify trends. Anonymity and confidentiality were ensured as all data were de-identified prior to analysis. Ethical approval and a request for waiver of informed consent for this retrospective study were obtained from the Stellenbosch University Health Research Ethics Committee (HREC Reference No: X20/04/017).

## Results

### Specialist surgical workforce profile

**Figure 1:**
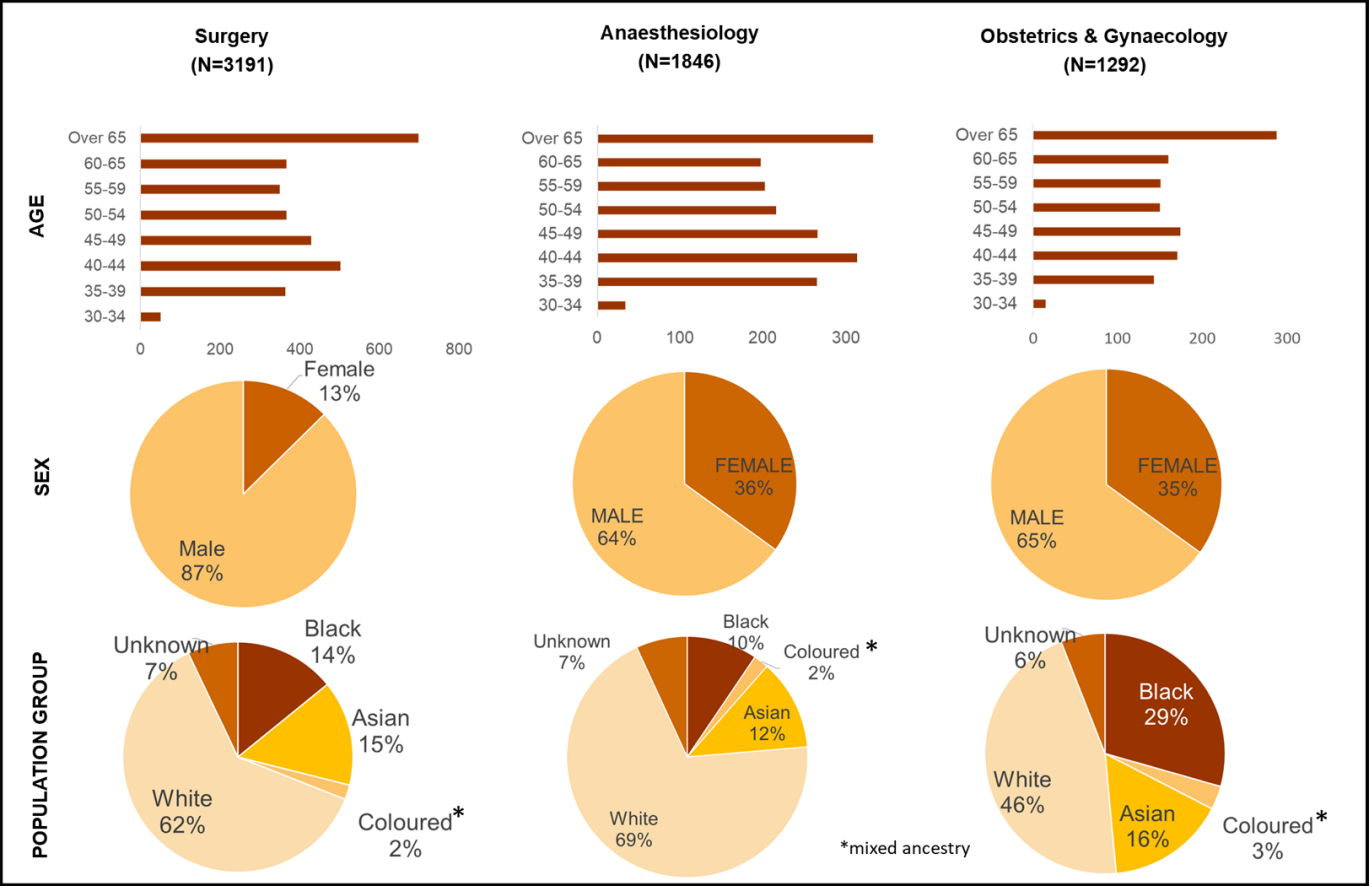
Demographic Profile of the Specialist Surgical Workforce in South Africa (2019).

As of December 2019, on the HPCSA register, there were 6329 specialists in the surgical workforce in South Africa including 3191 surgeons (50.4%), 1292 (20.4%) OBGYN, and 1846 (29.2%) anaesthesiologists. There were 4804 (75.9%) males and 1525 (24.1%) females. Over half of the registered surgical workforce was between 40–60 years of age (surgeons-52.7%, anesthesiologists-54.6% and OBGYN-51.6%) and formerly classified as white (surgeons-61%, anaesthesiologists-69.4% and OBGYN-45.5%). The majority of the surgical workforce was located in the three most densely populated and urbanised provinces, Gauteng (39.9%), Western Cape (25.4%) and KwaZulu-Natal (17.9%). In contrast, Eastern Cape (5.7%), the Free State (4.2%), Mpumalanga (2.4%), North West (2.1%), Limpopo (1.7%) and the Northern Cape (0.6%) had the lowest numbers of surgical workforce specialists. ***Figure 1*** provides a summary of the data according to age, sex, and population group.

### Specialist surgical workforce density

The specialist surgical workforce density for South Africa in 2019 was 10.5 per 100,000 population. The surgeon density was 5.3. This included a general surgeon density of 1.40 and an orthopaedic surgeon density of 1.60. Other sub-specialist densities are listed in ***Table 2***. In South Africa, regional hospitals typically only have general surgeons and orthopaedic surgeons whereas tertiary hospitals will have other sub-specialist surgeons.

**Table 2:** Specialist Surgical Workforce Density per 100,000 population in South Africa (2019).


SPECIALITY/SUB-SPECIALITY	SURGICAL WORKFORCE DENSITY

General Surgery	1.40

Vascular Surgery	0.11

Paediatric Surgery	0.05

Trauma Surgery	0.05

Gastroenterology	0.10

Ophthalmology	0.93

Plastic and Reconstructive Surgery	0.39

Orthopaedic Surgery	1.60

Cardiothoracic Surgery	0.24

Neurosurgery	0.40

Obstetrics and Gynaecology	2.16

Anaesthesiology	3.03


**Figure 2:**
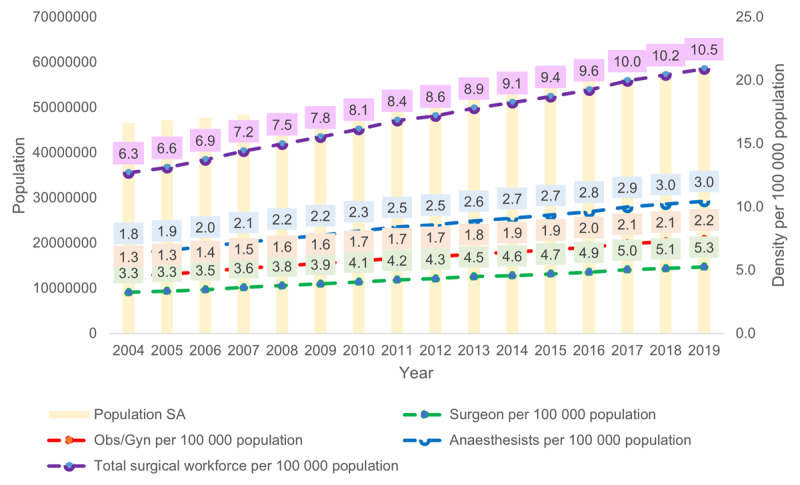
South African specialist surgical workforce density from 2004–2019.

**Figure 3:**
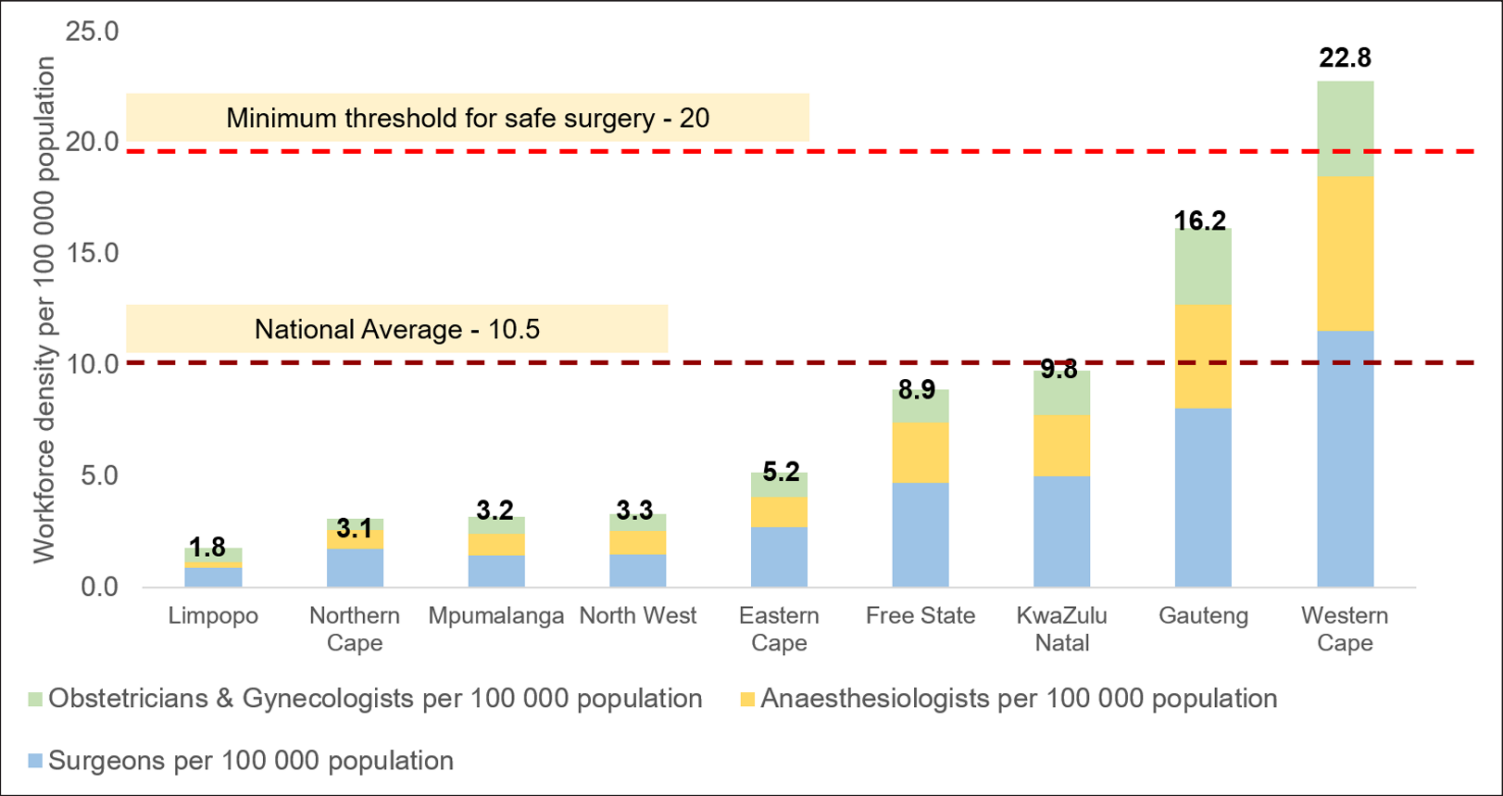
South African surgical workforce density by province (2019).

The anaesthesiologist density was 3.0 and the OBGYN was 2.2 (***Figure 2***). This varied between provinces from 1.8 (Limpopo) to 22.8 (Western Cape) per 100,000 (***Figure 3***).

### Surgical workforce trends from 2004–2019

**Figure 4:**
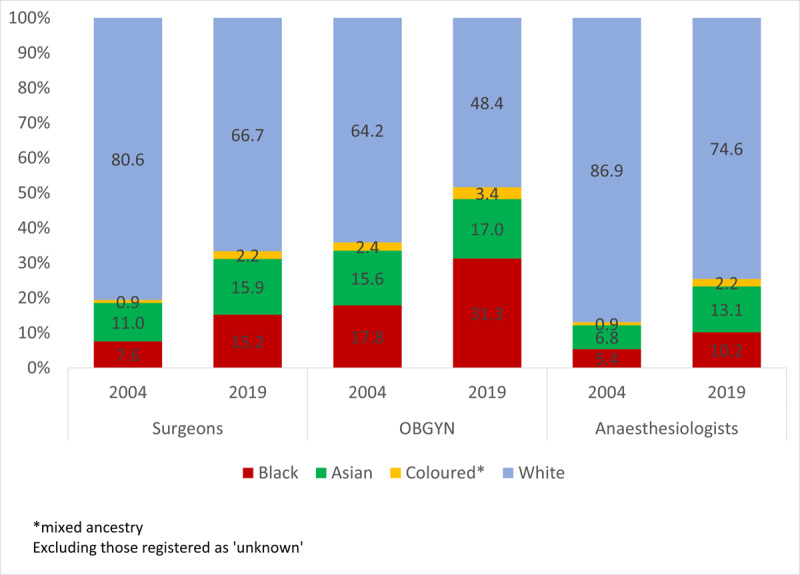
Distribution of the specialist surgical workforce by population group between 2004 and 2019.

**Figure 5:**
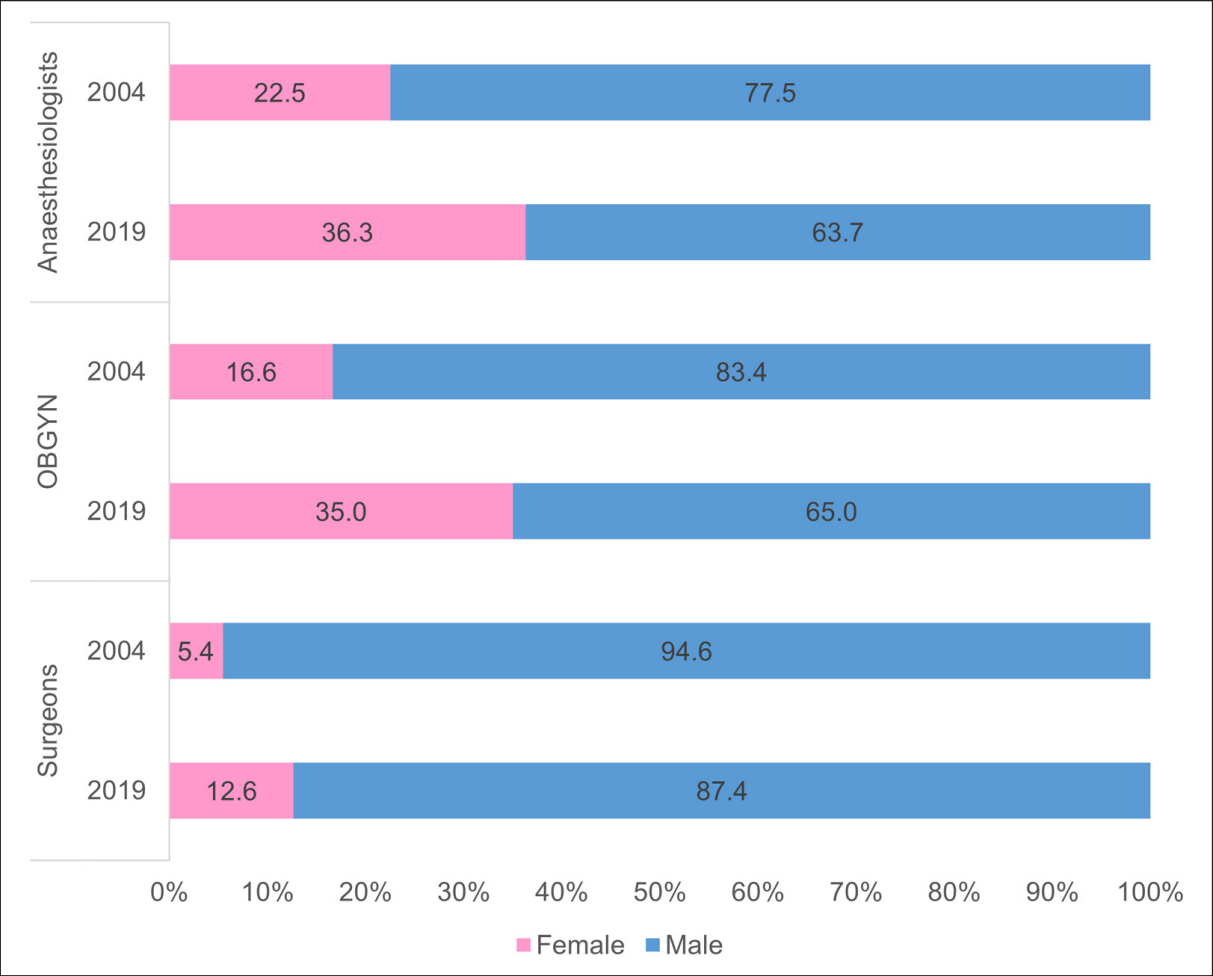
Distribution of females in the specialist surgical workforce between 2004 and 2019.

The specialist surgical workforce doubled from 2004 (N = 2956) to 2019 (N = 6144) with relatively similar average annual increases amongst surgeons (4.9%), OBGYN (5.2%), and anaesthesiologists (5.0%). The specialist surgical workforce density increased over the 15-year period from 6.3 in 2004 to 10.5 in 2019. The specialist density per 100,000 population between 2004 and 2019 increased from 3.3 to 5.3 for surgeons, 1.8 to 3.0 for anaesthesiologists and 1.3 to 2.2 for OBGYN (***Figure 2***). The specialist surgical workforce grew across all population groups at each five-year interval (***Figure 3***). The proportion of females within each cadre as well as those classified as other than white grew between 2004 and 2019 (***Figures 4*** and ***5***).

In 2004 females constituted 5.4% of surgeons compared to 12.6% in 2019. Similarly, in 2004, females constituted 16.6% of OBGYN compared to 35% in 2019. In 2004 there were 22.5% females in anaesthesiology compared to 36.3% in 2019.

## Discussion

Perioperative mortality in Africa is twice the global average [18]. Quality of and access to surgical care can only improve if and when there is a sufficient and adequate surgical workforce. This study found that the specialist surgical workforce density in South Africa (SA), an upper middle-income country (UMIC), was 10.5 per 100,000 persons which falls short of the LCGS minimum standard of 20 per 100,000 for safe surgery and is lower than several other UMIC such as Peru (41.77), Mauritius (34.57), Colombia (20.33), Maldives (15.96), and Malaysia (15.58) [1,19].

Other studies have shown that a higher workforce density correlates with an increased quality of care, specifically maternal survival [1]. An inadequate workforce density will also affect the minimum surgical volume needed to adequately address the burden of surgical disease [1].

SA is one of the most unequal countries in the world with a high Gini coefficient of 0.63 in 2015 and has a dual health care system (private and public healthcare facilities) with a diverse and historically racialized demographic profile [20,21]. We found that the distribution of the surgical workforce was also highly inequitable with a specialist workforce density in Western Cape, an urban and densely populated province, nearly twenty times higher compared to Limpopo, a rural sparely populated province. Improving the distribution of surgeons, anaesthesiologists, and OBGYN in South Africa between the private and public sectors is also important. A previous study showed that only 42% general surgeons work in the public sector which serves 86% of the population [10]. Further studies are needed to know if these maldistributions directly affect access to surgical care and ultimately clinical outcomes such as higher mortality from surgical conditions.

It is worth noting that the Eastern Cape and Western Cape have an almost similar population size, yet Western Cape has four times the density of surgeons, obstetrics and gynaecologists and anaesthesiologists (per 100,000 population) than Eastern Cape. Although North West has 60% of the Western Cape’s population, the density of surgeons, obstetrics and gynaecologists and anaesthesiologists (per 100,000 population) is 14% of that of the Western Cape.

While there is a shortage of surgical specialists, human resources for surgical healthcare in South Africa are on an upward trajectory. This is in line with the 2020 Human Resources for Health policy which emphasised the training and promotion of all appropriate and equitably distributed health workforce [22]. The surgical workforce has doubled over 15 years while the South African population increased by 26%. In addition, the demographic profile is becoming more equitable. Surgery, anaesthesia, and OBGYN have traditionally been dominated by male physicians and previously classified as white who constitute less than 8% of the total population. The racialized and gender gaps are, however, closing. The section of the population classified as Black is most under-represented in all three professions. For instance, in the year 2004, 5.4% anaesthesiologists, 7.6% surgeons and 17.8% OBGYN belonged to the category classified as the Black population who comprise almost 80% of the population of the country [23]. In over one and a half decade (in 2019), these numbers changed to 10.2% anaesthesiologists, 15.2% surgeons and 31.3% OBGYN identified themselves as black population group however the SA’s black population rose to 80.7% [24]. Recruiting from the other population groups can help increase the diversity and size of specialist surgical workforce. Ethnic concordance between patients and their physicians has also been shown to improve patient satisfaction [25].

Another level of inequity exists between public and private sectors, as per Wishnia et al., in 2019, there were 0.64 anaesthesiologists per 100,000 population in public sector and 9.69 in private sector and 0.62 OBGYN in public sector vs 6.57 in private sector [26]. Thus, South Africa is way below other middle-income countries in terms of medical specialists [26].

South Africa has rolled out National Health Insurance (NHI) which strives for universal health coverage. Our study found that majority of the surgical workforce is mid to late career. With a large proportion of these specialists predicted to retire in the next 10–15 years, more research into forecasting the needed numbers and a plan on how to expand post-graduate specialist surgical workforce education are needed.

This study has several limitations. Our analysis was based on a specific indicator proposed by the Lancet Commission on Global Surgery- specialist surgical workforce density. The specialist surgical workforce (surgeons, anaesthesiologists, and obstetrician/gynaecologists) are an essential cadre in surgical delivery. However, we acknowledge that without other cadres, such as nurses, mid-level providers, and non-surgeon physicians (such as family physicians), surgical delivery could not occur. Even the LCGS has realised that measuring the specialist surgical workforce underestimates the true surgical workforce in most LMICs where non-specialists provide essential surgical care and has proposed an updated metric with other cadres included as well [27]. Measuring their densities is difficult because we do not have an accurate way to capture which of these providers are contributing to surgical care in South Africa (i.e., not all family physicians provide surgical care and not all nurses are perioperative nurses).

The specialist surgical workforce density underestimates the true number of surgical providers because in South Africa non-specialists also provide surgical, anaesthesia, and OBGYN care. For example, Dell et al., reported there were three times the number of non-specialists providing general surgery care in 2014 compared to specialist general surgeons in South Africa [10]. Several rural generalist surgeons and family physicians are as well trained to provide surgical care in the district health system of South Africa. Surgery is a clinical domain in the training of South African family physicians [28]. Family physicians have a responsibility for access to, and the quality of, surgical services in their districts along with working with specialists and subspecialists at higher-level hospitals [29,30,31]. District hospitals within South Africa can serve as adequate training sites for family physicians in nationally expected skills in surgery, obstetrics and anaesthetics [32,33]. Thus, the family physician, especially in rural areas, may help meet the need for trained workforce in obstetric, anaesthetic and surgical skills [34,35]. Additionally, to meet the shortage of specialists further specialised courses such as nurse practitioner in midwifery (NPM) can be adopted (for the nursing professionals) along with strengthening nursing teaching [36]. Using HPCSA registration to identify the specialist surgical workforce could have led to overestimation if some practitioners emigrated (those registered in a province but have migrated), retired, or died [37].

## Conclusion

However, this study has contributed novel findings by describing the current gap and past trends in the South African specialist surgical workforce using a national physician’s database. South Africa falls short of the minimum specialist workforce density of 20 per 100,000 needed to provide adequate essential and emergency surgical care. In order to address the current and future burden of disease treatable by surgical care, South Africa needs a robust surgical healthcare system with adequate human resources, to translate healthcare services into improved health outcomes.

## Key questions

What is already known?

South Africa is an upper middle-income country with inequitable access to healthcare. There is a maldistribution of doctors between the private and public sectors, the latter which serves 86% of the population but has less than half of the human resources.

What are the new findings?

The study provides a comprehensive and detailed summary of the current surgical workforce in terms of HRH within South Africa. South Africa falls short of the minimum specialist workforce density of 20 per 100,000 needed to provide adequate essential and emergency surgical care.

What do the new findings imply?

While there is a shortage of surgical specialists, human resources for surgical healthcare in South Africa are on an upward trajectory. In order to address the current and future burden of disease treatable by surgical care, South Africa needs a robust surgical healthcare system with adequate human resources, to translate healthcare services into improved health outcomes.
